# Intraventricular Hemorrhage in Very Preterm Infants: A Comprehensive Review

**DOI:** 10.3390/jcm9082447

**Published:** 2020-07-31

**Authors:** Vianney Gilard, Abdellah Tebani, Soumeya Bekri, Stéphane Marret

**Affiliations:** 1 Department of Pediatric Neurosurgery, Rouen University Hospital, 76000 Rouen, France; vianney.gilard@chu-rouen.fr; 2 Department of Metabolic Biochemistry, Rouen University Hospital, 76000 Rouen, France; abdellah.tebani@yahoo.com; 3 Normandie University, UNIROUEN, CHU Rouen, INSERM U1245, 76000 Rouen, France; stephane.marret@chu-rouen.fr; 4 Department of Neonatal Pediatrics, Intensive Care and Neuropediatrics, Rouen University Hospital, 76000 Rouen, France

**Keywords:** intraventricular hemorrhage, germinal matrix, preterm, neonates, post-hemorrhagic hydrocephalus

## Abstract

Germinal matrix-intraventricular-intraparenchymal hemorrhage (GMH-IVH-IPH) is a major complication of very preterm births before 32 weeks of gestation (WG). Despite progress in clinical management, its incidence remains high before 27 WG. In addition, severe complications may occur such as post-hemorrhagic hydrocephalus and/or periventricular intraparenchymal hemorrhage. IVH is strongly associated with subsequent neurodevelopmental disabilities. For this review, an automated literature search and a clustering approach were applied to allow efficient filtering as well as topic clusters identification. We used a programmatic literature search for research articles related to intraventricular hemorrhage in preterms that were published between January 1990 and February 2020. Two queries ((Intraventricular hemorrhage) AND (preterm)) were used in PubMed. This search resulted in 1093 articles. The data manual curation left 368 documents that formed 12 clusters. The presentation and discussion of the clusters provide a comprehensive overview of existing data on the pathogenesis, complications, neuroprotection and biomarkers of GMH-IVH-IPH in very preterm infants. Clinicians should consider that the GMH-IVH-IPH pathogenesis is mainly due to developmental immaturity of the germinal matrix and cerebral autoregulation impairment. New multiomics investigations of intraventricular hemorrhage could foster the development of predictive biomarkers for the benefit of very preterm newborns.

## 1. Introduction

Germinal matrix-intraventricular-intraparenchymal hemorrhage (GMH-IVH-IPH) remains a serious complication in very preterm children born before 32 weeks of gestation (WG), and is particularly frequent in extremely preterm children born before 27 WG [1,2,3]. In the French cohort EPIPAGE 2 (Etude Epidémiologique sur les Petits Ages Gestationnels 2) [4], approximately 20 to 30% of infants with a gestational age below 29 WG present with GMH-IVH-IPH, corresponding in the United States to 12,000 cases each year [5]. One-third of neonates with GMH-IVH-IPH develop post-hemorrhagic hydrocephalus (PHH) and 10 to 20% require shunt insertion [6,7]. The onset of PHH is mainly determined by IVH grading [8], ranging from subependymal hemorrhage (grade I) to IVH with ventricular dilatation and IPH (grade IV). Improvements in obstetric care have led to an increase in survival of preterm infants secondary to the antenatal administration of corticosteroid and magnesium sulfate with a proven efficacy in reducing cerebral palsy (CP) [9,10,11]. Nevertheless, in cases of severe GMH-IVH-IPH, 40% of the preterm infants die from a neurological cause [5]. The association of prematurity, GMH-IVH-IPH and PHH is a strong determinant of impaired neurodevelopmental outcomes [12,13,14]. In the last 10 years, the incidence of preterm children with GMH-IVH-IPH has increased, likely due to the increased survival of extremely preterm children [5]. In a few cases, GMH-IVH-IPH can occur in fetuses during pregnancy or in children born at term. A correlation has been established between low gestational age at birth and the incidence and severity of GMH-IVH-IPH [15,16,17]. A better understanding of the pathophysiology of GMH-IVH-IPH and the role of neonatal angiogenesis in relation to the germinal matrix is essential to develop strategies to prevent and treat their complications.

The pathogenic mechanisms of GMH-IVH-IPH remain poorly understood, and neuroprotection measures are heterogeneous and sometimes controversial. As a result, there is little consensus on contemporary practices for the optimal management of these events.

For this review, an automated clustering approach has been applied to allow efficient filtering as well as topic clusters identification. The presentation and discussion of these clusters provide a comprehensive overview of existing data on the pathogenic mechanisms, complications, neuroprotection and biomarkers of GMH-IVH-IPH in very preterm children. This review highlights the need to identify reliable indicators of GMH-IVH-IPH onset and severity.

## 2. Experimental Section

### 2.1. Literature Analysis

In this study, we performed a programmatic literature search for a more efficient and reproducible review process using the Adjutant R package [18]. We searched for articles related to intraventricular hemorrhage in preterms that were published between January 1990 and February 2020. We used two queries ((Intraventricular hemorrhage) AND (preterm)). The resulting document corpus included PubMed IDs, year of publication, authors, article titles, article abstract and any associated Medical Subject Heading (MeSH) terms. Titles and abstracts were decomposed into single terms, stemmed and filtered by the Adjutant package. The term frequency–inverse document frequency metrics for each term were used to create a sparse document-term matrix (DTM) for further analysis. *t*-Distributed Stochastic Neighbor Embedding (*t*-*SNE*) [19] and hdbscan [20] algorithms were used to perform unsupervised clustering using DTM data. The coordinates generated by t-SNE were used in the hdbscan algorithm to derive the topic clusters. Each cluster was then assigned a topic by using the five most frequent terms within the cluster.

### 2.2. Manual Curation: Inclusion and Exclusion Criteria

Following the topic clustering step, we validated our clusters using external manual curation, assessing the correspondence between articles and cluster topics. Each sampled article was examined and either considered acceptable for further analysis or rejected. Inclusion criteria were topic relevance, article in English, human data, original research or clinical trial. We further refined corpus and cluster naming. Table S1 contains a list of all the articles with their corresponding cluster.

## 3. Results 

### 3.1. Literature Mining and Topic Clusters

**Figure 1:**
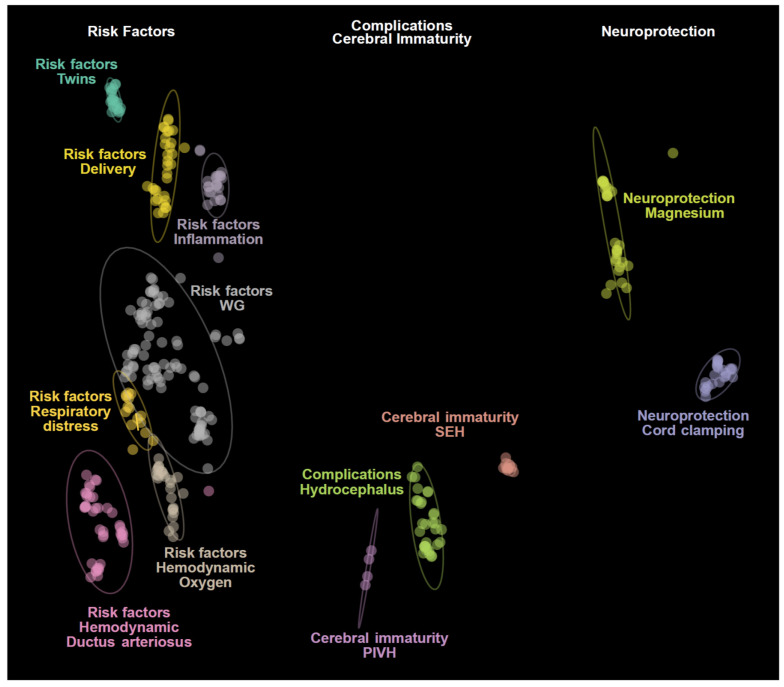
Topic representation of the included literature related to preterm birth and intraventricular hemorrhage. The figure highlights 12 clusters spread over three main groups: risk factors, complications and therapeutics of neonatal intraventricular hemorrhage. PIVH: Periventricular/intraventricular hemorrhage. SHE: Subependymal hemorrhage.

The first-round analysis generated a document corpus of 1093 articles related to intraventricular hemorrhage and preterms published in the past 30 years (Table S1). Using article titles and abstracts, we derived topic clusters in an unsupervised manner, and classified articles as either belonging to a named topic cluster or not belonging to a cluster. Articles that never formed part of a cluster were removed from further analysis, leaving 353 documents that formed 12 clusters. Clusters were assigned topics via the five most frequent terms within the cluster and manual curation of the included articles. These results revealed that intraventricular hemorrhage in preterm child literature is primarily structured around (i) pathogenesis, (ii) risk factors such as twinning, delivery, prematurity, inflammation and hemodynamic or respiratory instability, (iii) complications and (vi) neuroprotection (Figure 1). The full list of articles and related clusters are presented in Table S1.

### 3.2. Pathogenesis of GMH-IVH-IPH

The determinants of GMH-IVH-IPH in preterm children are multiple, essentially based on alteration of the germinal matrix and impairment of cerebral autoregulation. Different factors underlying these alterations have been described, such as coagulation disorders and genetic traits.

#### 3.2.1. Immaturity of the Germinal Matrix 

The germinal matrix (GM) is located deep in the brain, in the caudothalamic groove. The GM is a highly functional metabolic region with intense angiogenesis compared to the cerebral cortex at this stage [21]. The angiogenesis of the GM is promoted by the in-situ secretion of vascular epithelial growth factor (VEGF) and angiopoietin (ANGPT) [22]. Moreover, a placenta–brain angiogenesis axis has been described [23]. Placental growth factor (PlGF), solely produced by human placenta, is involved in GM angiogenesis and acts on the angiogenic flt-1 receptor (VEGFR-1) after penetration in the fetal brain [23]. It has been shown that the subventricular zone of the lateral ventricles is implicated in neurogenesis. Neurogenesis is regulated by the proliferation of endothelial cells with a spatial correlation between neural progenitor cell migration and vascular growth [21,24,25]. The specificity of cerebral vessels is to form a blood–brain barrier (BBB) complex interface between the brain parenchyma and the endothelial cells [26]. Its role is to extract vital molecules for the brain and prevent toxic substances from altering the brain functions. The BBB is composed of endothelial cells, tight junctions, basement membrane proteins and astrocyte end feet [27]. Thus, in the case of preterm birth, the alteration of BBB components increases its permeability, causing the crossing of toxic substances into the brain and a higher propensity for bleeding [28]. The perivascular coverage of glial fibrillary acidic protein (GFAP) in the GM compared to the brain cortex may be a factor of vascular fragility [29]. Fibronectin and collagen are necessary for blood vessel stabilization. In cases of prematurity, the fibronectin level is lower in the GM, and collagen chain expression increases with gestational age [30]. Similarly, basal membrane, tight junction and astrocyte end foot expression are altered by a low gestational age at birth [31]. All these data emphasize the impact of gestational age on cerebral vessel organization. The impairment of angiogenesis comes with the alteration of neonatal neurogenesis [32]. The occurrence of bleeding in preterm children is associated with limited cell death and the suppression of cell proliferation with lasting effects on astrocytogenesis and oligogenesis [33,34]. Neuronal degeneration following intraventricular hemorrhage, and particularly neuronal death in the hippocampus, may be due to hemoglobin pro-apoptotic properties through the up-regulation of Jun N-terminal kinases (JNK) and CD163 [35]. Other factors have been reported as implicated in the modification of BBB permeability such as tissue plasminogen activators [36,37], P-glycoprotein (P-gp) [38] or the overexpression of N-methyl D-aspartate (NMDA) receptors [39]. It has been shown in animal models that the antenatal administration of corticosteroids has an impact on the BBB via the inhibition of VEGF-induced proteases, leading to a decreased risk of GMH-IVH-IPH [40].

#### 3.2.2. Disruption of Cerebral Homeostasis 

In this context of cerebral immaturity, it has been proven that the etiology of GMH-IVH-IPH is multifactorial and all risk factors of prematurity and oxidative stress such as infection, twinning, placental retro hematoma and cord clamping are responsible for disruption of cerebral homeostasis and blood flow [41]. Cerebral blood flow (CBF) provides the oxygen and energy necessary for neuronal metabolism. CBF is finely regulated by intravascular pressure, length of the vessel and vascular resistance [42]. The ability of the organism to maintain physiological cerebral perfusion despite CBF fluctuations is called cerebral autoregulation. Several techniques have been used to explore CBF and its regulation in neonates: xenon clearance, transcranial doppler and regional oxygenation by near-infrared spectroscopy (NIRS) [43]. In preterm infants, cerebral autoregulation has been found to be altered compared to neonates born at term [44]. Thus, in preterm infants, when cerebral autoregulation is altered, the mean arterial blood pressure remains low despite cerebral hypoperfusion [45]. Furthermore, at birth, there is a transfer from fetal to postnatal circulation with an acute increase in vascular resistance. In premature neonates, this vascular stress is poorly compensated [46], as illustrated in the case of chorioamnionitis, the subsequent inflammation, which could be associated with prolonged premature rupture of membranes (PPROM) [47]. Patent ductus arteriosus is a frequent condition in preterm births. In this case, the existence of a blood shunt between pulmonary and systemic circulation results in increased left cardiac ventricle output and decreased vascular resistance leading to cerebral hypoperfusion [48]. Another determinant of CBF is pH fluctuation. In cases of respiratory distress at birth, positive pressure ventilation and metabolic acidosis can cause variations in systemic blood flow [49]. Hence, all these factors are responsible for intense hemodynamic fluctuations in the first days of life and lead to altered cerebral autoregulation. This phenomenon has a strong impact on the GM, which is fed by the distal arterial vascularization, and is therefore vulnerable to low blood flow and ischemia [50].

#### 3.2.3. Coagulation Impairment 

Coagulation disorders have been described in the preterm neonates but remain debated. Nevertheless, prematurity comes with thrombocytopenia, which is an independent factor of the presence and extent of bleeding compared to children without thrombocytopenia [51]. Frequent causes of early thrombocytopenia are alloimmunization [52] or chronic fetal hypoxia secondary to pre-eclampsia [53]. The onset of late thrombocytopenia (>72 h after birth) is mainly related to an inflammatory state. Sepsis is usually associated with an increase in thrombopoietin levels to compensate for platelet consumption due to an inflammatory condition. This process appears to be impaired in premature neonates, which explains their inability to recover their platelet pool quickly enough following platelet consumption. The small size of megakaryocytes, precursors of platelets, in neonates is also a factor of decreased platelet production [52]. Thus, the mechanism of neonatal thrombocytopenia combines platelet destruction and low platelet production. The determinants of neonatal thrombocytopenia are imperfectly understood. Coagulation disorders are found in preterm infants with IVH with abnormal levels of INR (International Normalized Ratio), antithrombin III and fibrinogen [54]. Streif et al. studied the level of factor VIIa and its effects on thrombin from blood samples in preterm children, children born at term and adults. They found that the effects of factor VIIa on factor II in preterm children were more pronounced and could lead to an increased risk of bleeding [55].

#### 3.2.4. Genetic Factors 

The genesis and onset of neonatal GMH-IVH-IPH are most likely underpinned by genetic factors, which in turn can be modulated by environmental factors [41]. Genetic explorations involve vascular organization, inflammation and coagulation disorders. At the GM level, COL4A1, a gene coding for procollagen type 4, plays a key role in vessel stabilization. Alterations in this gene are associated with neonatal intracerebral hemorrhage in preterm infants [56,57]. Hence, mutations of the COL4A1 gene cause perforation of the basement membrane of the vascular wall responsible for GM hemorrhage. It has been shown that pathogenic variants in the gene NOS3 coding for endothelial nitric oxide synthase (eNOS) are associated with a 3- to 4-fold higher risk of bleeding and have an impact on cerebral autoregulation in neonates [58]. Endothelin-1 is a vasoconstrictor, and its role in strokes has been described in a mouse model of strokes. Variants in the endothelin 1 (END1) gene play an unclear role in the impairment of cerebral autoregulation in neonates [58,59]. Other gene dysregulations involved in the onset of IVH have been reported. Variants in proinflammatory interleukin genes such as Il-1ß or tumor necrosis factor genes are associated with IVH in preterm neonates. The level of proinflammatory cytokines in the amniotic fluid is correlated with the occurrence and severity of white matter lesions and cerebral palsy [60]. Lastly, genes coding for coagulation have been shown to be altered in a context of prematurity. Indeed, the variants factor V Leiden or prothrombin G20210A were reported to be predictive of neonatal IVH [61]. In summary, it is undeniable that genetic alterations have an impact on the onset of IVH, but the understanding of the different pathways is unclear. 

### 3.3. Complications of GMH-IVH-IPH

GMH-IVH-IPH could be associated with ventricular dilatation and diffuse parenchymal white matter lesions (i.e., diffuse gliosis and/or periventricular cystic lesions) visualized by cranial ultrasound and cerebral MRI. These complications may result in brain atrophy [12]. Due to the impaired resorption of cerebrospinal fluid (CSF), GMH-IVH-IPH is responsible for PHH in up to 15% of preterm children [1]. The diagnosis, timing and type of shunt used in PHH are much debated in the literature but mostly based on ventriculosubgaleal shunt or ventricular tapping followed by ventriculoperitoneal shunt [17,62]. However, it is commonly accepted that the onset of PHH and the need for a shunt insertion is an independent risk factor of impaired development in a child [13]. Moreover, the shunt exposes this group of children to increased morbidity and mortality due to the risk of a shunt dysfunction and recurrent surgery exposition [5,63]. The rate of death and cerebral palsy in older children is correlated with the grading of GMH-IVH-IPH, gestational age and weight at birth and the associated complications of preterm birth [13]. Due to the high risk of complications in preterm children with GMH-IVH-IPH, their early detection is mandatory to enable appropriate neuroprotective management. 

### 3.4. Neuroprotection

Antenatal steroid therapy, as well as antenatal administration of magnesium sulfate in women at risk of very preterm birth, have been associated with a decrease in the rate of GMH-IVH-IPH and therefore limit the risk of death or cerebral palsy in survivors [64,65]. The impact of these neuroprotective agents appears to be major before 33 WG and is therefore widely practiced. Both magnesium sulfate and corticosteroids could provide a benefit by (i) stabilizing the arterial blood pressure and CBF in preterm children and (ii) accelerating brain and pulmonary maturation through an anti-inflammatory effect [11]. Similarly, the treatment of hypotension with fluids and the administration of vasoactive agents at birth have been tested in preterm infants to restore the CBF balance. Nevertheless, a Cochrane review could not conclude a benefit of volume expansion in these children [66]. Indomethacin or ibuprofen was proposed to prevent brain injury. Although indomethacin is of interest to prevent patent ductus arteriosus, its impact on neurodevelopment was not clarified by recent studies [67]. The use of vitamin E was also proposed with discordant results in preterm children with GMH-IVH-IPH and is not in current use [68]. A randomized trial on the administration of erythropoietin was performed and concluded an absence of benefit on child neurodevelopment [69]. Obstetric care is also of importance in the prevention and management of preterm birth complications. As a matter of fact, a higher rate of severe GMH-IVH-IPH has been associated with umbilical cord milking compared to delayed umbilical cord clamping, suggesting that a change in systemic blood flow with umbilical cord milking may be transferred to the CBF [70]. Nevertheless, a Cochrane review concluded an insufficiency of data to support a clear timing of cord clamping [71]. Moreover, in extremely preterm neonates, postnatal nursing interventions (positioning the head in the middle position, elevating the head of the incubator to facilitate cerebral venous outflow, avoidance of elevation of legs during diaper change, slow arterial/intravenous flushing) decreased the risk of GMH-IVH-IPH in a “before-after” study [72,73].

### 3.5. Biomarkers

A better understanding of the physiopathology of GMH-IVH-IPH could pave the way to predictive biomarkers of this complication. As reported by Yang et al., the overexpression of VEGF level in umbilical cord blood of preterm neonates could be an indicator of GMH-IVH-IPH risk [40]. Other predictive parameters have been described, such as early blood gas characteristics: severe metabolic acidosis during the first 72 h of life is associated with an increased risk of GMH-IVH-IPH in preterm children < 29 WG. The protein S100B, expressed in glial cells, is proposed as an early neurobiomarker of intracerebral bleeding and GMH-IVH-IPH in neonates. The S100B level is increased in several biological fluids, such as CSF, saliva and, with high specificity, in cord blood [74,75]. Other possible biomarkers are vasoactive agents such as adrenomedullin (AM), a vasodilatator peptide. The circulating AM level was elevated in preterm patients presenting GMH-IVH-IPH, suggesting that it may be secreted in response to altered cerebral blood circulation [76]. Various potential biomarkers are being investigated, such as neuron-specific enolase, activin A or glial fibrillary acidic protein [74,77,78].

### 3.6. A New Era: Omics-Based Approaches

Over the last decade, the rise of omics techniques has opened a new era in the diagnosis and understanding of diseases in different fields, shaping the so-called new discipline of Precision Medicine [79,80]. These techniques allow for a comprehensive overview of the altered biological processes by interrogating different biosamples such as CSF, urine, blood or tissue depending on the pathology. Precision medicine can provide valuable tools for the development of biomarkers and innovative therapies. Intraventricular hemorrhage in preterm neonates is no exception, and several pathways are already proposed to enhance molecular understanding of prematurity [81,82,83]. A targeted metabolomics approach study using liquid chromatography–mass spectrometry in urine samples of preterm neonates showed clear differences between preterms presenting either with or without GMH-IVH-IPH. A total of 20 metabolites were differentially expressed between groups and appeared as strong predictors of GMH-IVH-IPH onset, especially at day 1 [84]. Omics approaches in preterm hemorrhage are still rare but seem to be promising and could circumvent the pitfalls of reductionist classical approaches.

## 4. Limits

The limitations of this review are mainly related to the complexity of GMH-IVH-IPH and the fact that it represents only one of the complications of prematurity. The impact of other preterm birth determinants, including prematurity itself, metabolic impairment and infectious diseases, may hamper the pathophysiology understanding and the biomarker interpretation. Moreover, the data presented in the related studies are highly heterogeneous since ethical frameworks and neonatal care practices diverge between different centers and countries, representing, thus, an obvious analytical bias. Finally, due to the difficulty and cost of conducting large studies, these results are often originated from single-center studies with limited numbers of children included. There is a clear and urgent need for newer, multimodal and more comprehensive approaches.

## 5. Conclusions

The incidence of GMH-IVH-IPH in preterm children remains high despite research on this topic and preventive measures. Clinicians should consider that the pathogenesis of GMH-IVH-IPH is mainly due to alteration of the germinal matrix, impairment of cerebral autoregulation, coagulation disorders and genetic factors. The periventricular zone is a source of neurogenesis and astrocytogenesis, and the onset of bleeding is responsible for impaired development. Presently, despite improvements in the molecular understanding of GMH-IVH-IPH, the identified biomarkers have some drawbacks, and the therapeutic strategies derived from these data are disappointing [15,85]. New integrative multiomic approaches in intraventricular hemorrhage could foster the development of predictive biomarkers for the benefit of very preterm children.

## Supplementary Materials

The following are available online at https://www.mdpi.com/2077-0383/9/8/2447/s1, Table S1: Articles and clusters related to intraventricular hemorrhage and preterms published in the past 30 years.
